# IL4I1-driven AHR signature: a new avenue for cancer therapy

**DOI:** 10.1038/s41392-021-00529-z

**Published:** 2021-03-10

**Authors:** Zuli Wang, Tiansheng Li, Chao Mao, Wenliang Liu, Yongguang Tao

**Affiliations:** 1 NHC Key Laboratory of Carcinogenesis and Hunan Key Laboratory of Cancer Metabolism, Hunan Cancer Hospital and The Affiliated Cancer Hospital of Xiangya School of Medicine, Central South University, Changsha, Hunan China; 2 Key Laboratory of Carcinogenesis and Cancer Invasion of the Chinese Ministry of Education, Cancer Research Institute, Central South University, Changsha, Hunan China; 3 Department of Thoracic Surgery, Hunan Key Laboratory of Early Diagnosis and Precision Therapy in Lung Cancer, Second Xiangya Hospital, Central South University, Changsha, Hunan China

**Keywords:** Tumour immunology, Molecular medicine

**Fig. 1:**
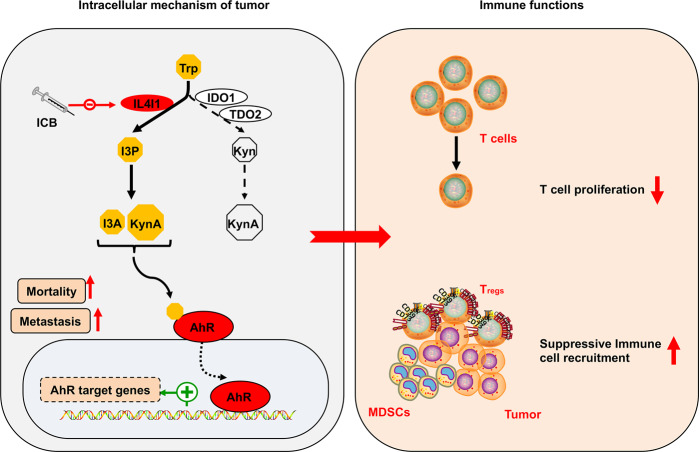
Newly identified IL4I1 which is induced by immune checkpoint blockade (ICB) mediates AHR signature genes through I3P-KynA/I3A metabolic pathway parallel to IDO1 and/or TDO2-driven AHR signaling. On one hand, IL4I1 can promote cancer cell motility and metastasis, on the other hand, it also inhibits T-cell proliferation and recruits suppressive immune cells

Aryl hydrocarbon receptor (AHR) was considered to be an important pan-tumor therapeutic target, but small molecule inhibitors targeting AHR target gene IDO1 have failed in clinical trials. The recent paper published in *Cell* by Opitz et al. explained the failure of previous clinical trials and identified new therapeutic targets^1^ (Fig. 1).

AHR is a ligand-activated transcription factor belonging to the basic helix–loop–helix–PER–ARNT–SIM (bHLH–PAS) subgroup of the bHLH superfamily of transcription factors.^2^ Among the ligands of AHR, endogenous tryptophan (Trp) is the most important one. The kynurenine (Kyn) pathway activated by indoleamine-2,3-dioxygenase 1/2 (IDO1/2) or tryptophan-2,3-dioxgenase (TDO2) is considered to be the main tryptophan metabolic pathway in humans, which can produce the AHR agonist Kyn and kynurenic acid (KynA). Interestingly, due to the activation of AHR, tumor cells express high levels of IDO1 and TDO2 to promote plasticity. Since the Kyn-AHR axis inhibits the functions of T cells, the inhibition of Trp-catabolic enzymes also restricts the occurrence and development of tumor. However, the first phase III clinical trial of small molecule inhibitors of IDO1 failed, suggesting that there were other resistance mechanisms against IDO1 inhibition. Interestingly, immune checkpoint blockade (ICB) induces IDO1 and L-amino acid oxidase IL4I1, and IDO1 inhibitors do not block IL4I1,^1^ indicating that inhibition of novel AHR-mediator IL4I1 is an approach to cancer treatment.

AHR targets change with cell, tissues, and ligands for the context specificity which hinders pan-cancer analysis of AHR activity. To overcome this difficulty, Opitz et al. combined gene expression datasets with natural language processing (NLP) yielding 166 AHR signature genes regulated by AHR across various cell types, diverse ligands, and different AHR agonists or inhibitors.

To assess whether IDO1 and TDO2 were indeed associated with AHR activity,^3^ Opitz et al. performed the high or low group of IDO1 or TDO2 expression and weighted gene co-expression network analysis (WGCNA) across the 32 TCGA tumor entities and found that there was a positive correlation between IDO1 or TDO2 and AHR signaling in 23 tumor types. IDO1 and TDO2 did not appear in the AHR-associated modules, but the L-amino acid oxidase IL4I1 exhibited the highest incidence instead of Trp-catabolic enzymes (TCEs). Interestingly, the classic function of IL4I1 is recognizing and catalyzing the oxidative deamination of phenylalanine (Phe) into phenylpyruvic acid (PP) accompanied by the generation of H_2_O_2_ and NH_3_, indicating that IL4I1 was a novel potential target gene of AHR. Next, they treated glioblastoma (GBM) cells with IL4I1-derived metabolites, which increased the nuclear localization of AHR and enhanced AHR target genes transcription. Moreover, supernatants of cells with IL4I1 expression suppressed T-cell proliferation. Importantly, high IL4I1 levels were associated with reduced survival in GBM patients.

To explore IL4I1-derived metabolites activate AHR, AHR-proficient cells were exposed to three metabolites including PP, hydroxyphenylpyruvic acid (HPP), and indole-3-pyruvic acid (I3P) that were converted from Phe, tyrosine (Tyr), and Trp by IL4I1.^4^ Surprisingly, the results showed that I3P significantly enhanced the expression of AHR target genes while PP and HPP did not influence their levels in multiple cell types. I3P did not only induce AHR nuclear translocation and transcription, but enhanced the motility of GBM cell and reduced CD8^+^ T cells proliferation in an AHR-dependent manner. Compared to the established AHR agonists Kyn and KynA, I3P induced AHR activity at lower concentrations, which signified that I3P represents a novel onco-metabolite. Moreover, the increase of KynA in the cell supernatant came from the metabolism of I3P. Among I3P-derived metabolites, indole-3-acetic acid (IAA) and indole-3-lactic acid (ILA), except indole-3-aldehyde (I3A), could not activate AHR, indicating that IL4I1-converted I3P produced KynA and I3A metabolites leading to AHR activation.

In the clinical significance of IL4I1, the authors found that IL4I1 expression and AHR activity were enhanced in primary cancer tissues and were higher in metastatic melanoma compared to primary melanoma. In fact, although IL4I1 drives the motility of tumor cells, immune modulation is the most enriched function of IL4I1. An accumulation of myeloid-derived suppressor cells (MDSCs) and T_regs_, a feature of chronic lymphocytic leukemia (CLL), which was strongly associated with high IL4I1 levels. Besides, IL4I1 expression had a positive correlation with AHR activity in patients with CLL. The authors, therefore, utilized aggressive CLL mouse model^5^ to test anti-tumor immunity effects of IL4I1. Compared to tumor-free mice, it turned out to be that *IL4i1* was among the most upregulated genes in tumor-supportive monocytes. Conversely, *IL4i1*^*−/−*^ chimeras mice reduced tumor burden, indicating the role of IL4I1 in immune escape. Subsequently, lack of IL4I1 mitigated CD8^+^ effector T cells (T_eff_) exhaustion through clonally expanded T_eff_ in TCL1 AT mice. Then, transcriptome analysis of sorted T_eff_ from tumor-bearing WT and *IL4i1*^*−/*−^ mice verified that IL4I1 deletion increased expression of T_eff_ genes and reduced *Ahr* levels, suggesting that lack of IL4I1 enhances CD8^+^ T-cell function. Last but not least, IL4I1 could develop resistance against ICB due to its immunosuppressive effects. The data showed that anti-PD1 monoclonal antibody nivolumab increased IL4I1 and IDO1 expression and resulted in AHR activation. In combination with anti-PD1 ICB, multiple IDO1 inhibitors that did not limit IL4I1 enzymatic activity has failed on therapy in phase III clinical trial. Together, IL4I1 developed a novel metabolic resistance mechanism against ICB and/or IDO1 inhibitors.

Collectively, combining gene expression analysis and NLP, Opitz et al. first discovered a pan-tissue AHR signature mediator IL4I1 that was linked with cancer cells migration and metastasis in two aspects: (i) IL4I1 does not only play the role in oxidative deamination, but also acts as a tryptophan metabolic enzyme. (ii) The pathway of activation of AHR by the metabolites of IDO1 and TDO2 enzymes is not a suitable immune checkpoint, but the I3A-AHR axis regulated by IL4I1 plays an important role. The new functions of IL4I1 on the recruitment of MDSCs and Tregs in tumor have also been explored. Based on the results that IL4I1 develops a metabolic resistance mechanism against ICB and/or IDO1 by activating AHR, this research provided a new and crucial avenue for cancer therapy.
